# Regression Model Fitting With Quadratic Term Leads to Different Conclusion in Economic Analysis of Washington State Smoking Ban [Response to Letter]

**Published:** 2010-12-15

**Authors:** Myde Boles, Clyde Dent, Julia Dilley, Julie E. Maher, Michael J. Boysun, Terry Reid

**Affiliations:** Multnomah County Health Department and Oregon Public Health Division, Portland, Oregon; Multnomah County Health Department, Portland, Oregon; Multnomah County Health Department and Oregon Public Health Division, Portland, Oregon; Multnomah County Health Department and Oregon Public Health Division, Portland, Oregon; Washington State Department of Health, Portland, Oregon; Washington State Department of Health, Portland, Oregon

## To the Editor:

We were interested to read Ma and McClintock's letter (1) about our analysis of taxable retail sales (TRS) data (2). Although we agree in general that different models of data can lead to different conclusions, we disagree that our analysis misrepresents those data. Rather, we find the reanalysis and presentation in their letter to be misleading.

Ma and McClintock reiterate our observation that the TRS data during this time do not follow a linear trend — with an obvious upturn in 2006 through 2007 and a smaller downturn in 2002 through 2003 — and question our use of a linear model to describe these data. In our analyses, we examined a segmented regression approach (3) to address this nonlinearity, using a theoretical break point at 2006 to delineate periods before and after passage of a smoke-free law (SFL) in Washington. Ma and McClintock put forth a linear model with a quadratic term to address the nonlinearity in these data, with no theoretical justification for the mechanism that would drive such a function. The use of a quadratic term suggests an exponential growth in TRS post-SFL, whereas we suggest a theory-based, more moderate, and flexible linear growth function due to the SFL. Although the figure presented by Ma and McClintock correctly represents their model, it misrepresents our model as a single straight-line fit when it actually has 2 linear segments (Figure).

**Figure 1:**
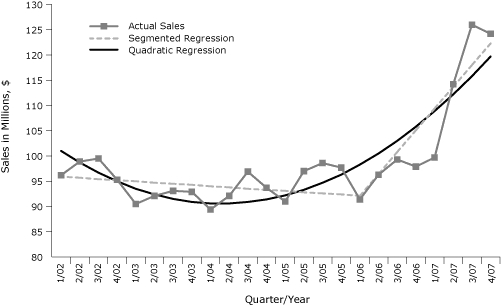
Segmented and quadratic regressions fit to taxable retail sales in bars and taverns in Washington State after the implementation of a smoke-free law, from the first quarter of 2002 (1/02) through the fourth quarter of 2007 (4/07). Values are adjusted for inflation to the Consumer Price Index (www.bls.gov/cpi/).

**Table:** 

**Quarter/Year**	**Sales in Millions, $**	**Segmented**	**Quadratic**
1/02	96.2	95.9	101.0
2/02	98.9	95.7	98.7
3/02	99.5	95.4	96.7
4/02	95.3	95.2	95.0
1/03	90.5	95.0	93.5
2/03	92.1	94.7	92.4
3/03	93.1	94.5	91.5
4/03	92.9	94.3	90.9
1/04	89.4	94.0	90.6
2/04	92.1	93.8	90.6
3/04	96.9	93.5	90.9
4/04	93.7	93.3	91.4
1/05	91.0	93.1	92.2
2/05	97.0	92.8	93.3
3/05	98.6	92.6	94.7
4/05	97.7	92.4	96.3
1/06	91.4	92.1	98.3
2/06	96.3	96.4	100.5
3/06	99.3	100.8	103.0
4/06	97.9	105.1	105.8
1/07	99.7	109.4	108.9
2/07	114.2	113.7	112.2
3/07	126.0	118.0	115.8
4/07	124.2	122.3	119.7

In addition, Ma and McClintock's contention that their model fits the data better, supported by an improved *R* ^2^ value, is incorrect. In fact, the competing models, adjusted only for inflation — one with a quadratic term to specify the post-SFL period and the other with a linear segmented term to model that period — have nearly the same *R* ^2^ value; our segmented model has a slightly higher value, suggesting a better fit (0.888 vs 0.875, respectively).

Ma and McClintock state that their results suggest that "the smoking ban did not affect the taxable sales revenue over the time." This statement is inaccurate in the context of the model they present. Rather, the quadratic term in their model and the post-SFL segment term in our model both provide evidence that TRS increased dramatically post-SFL.

Furthermore, Ma and McClintock present a table that is flawed. That table contains projected TRS post-SFL "with and without a quadratic term." The authors contend that the difference between projected and actual sales using a model with a quadratic term is large and opposite in sign to our estimated differences without that term. The principal error in this table is that although the "without quadratic term" column (our model projections) correctly reported the forecasted trend by using the model fit to the pre-SFL values (the first segment coefficient), the "with quadratic term" column actually uses the post-SFL predicted values as the base, as if the quadratic term applied to that interval, not to the values forecasted using a model fit to the data pre-SFL. In fact, it is because the quadratic term overpredicts "exponential growth" post-SFL that the residual values they present show the pattern they do. The comparison in that table, and the conclusions reached by Ma and McClintock, are incorrect.

We also note for clarity that the raw data table presented by Ma and McClintock do not agree completely with those we provided to them, possibly because they used a different inflation adjustment than the one we provided to them.

Given Ma and McClintock's misunderstandings and misrepresentations of our analysis, we stand firmly by our initial analysis and conclusions. As the authors state, we provided raw TRS data. In addition, we communicated with them about methods as an aid to their conducting a similar analysis with data from their state. Their letter to the journal is the first indication we received that they had concerns about our methods.
